# Retraction Note to: A new synthetic derivative of cryptotanshinone KYZ3 as STAT3 inhibitor for triple-negative breast cancer therapy

**DOI:** 10.1038/s41419-021-03809-2

**Published:** 2021-05-21

**Authors:** Wenda Zhang, Wenying Yu, Guiping Cai, Jiawen Zhu, Chao Zhang, Shanshan Li, Jianpeng Guo, Guoping Yin, Chen Chen, Lingyi Kong

**Affiliations:** State Key Laboratory of Natural Medicines, Department of Natural Medicinal Chemistry, China Pharmaceutical University, 24 Tong Jia Xiang, 210009 Nanjing, China

Retraction Note to: *Cell Death & Disease*

10.1038/s41419-018-1139-z published online 27 October 2018

The Editors have retracted this article. After publication concerns were raised about two of the figures. Specifically:

- In Figure 3B there appear to be areas of overlap and duplication
- In Figure 6C the HE T1 and HE T2 panels appear to be the same

The Editors therefore no longer have confidence in the data presented. Wenda Zhang, Wenying Yu, Guiping Cai, Jiawen Zhu, Shanshan Li, Jianpeng Guo, Guoping Yin, Chen Chen and Lingyi Kong disagree with this retraction. Chao Zhang has not responded to any correspondence from the Editors or the Publisher about this retraction.

